# Quantitative Consistency of Amide Proton Transfer-Weighted MRI for Brain Tumor Differentiation: Systematic Review of Clinical Evidence

**DOI:** 10.3390/tomography12050065

**Published:** 2026-05-06

**Authors:** Julius Juhyun Chung, Tianwen Ma, Phaethon Philbrook, Toby Zhou, Adam Ezra Goldman-Yassen, Phillip Zhe Sun

**Affiliations:** 1 Non-Human-Primate Imaging Center, Emory National Primate Research Center, Emory University, Atlanta, GA 30329, USA; julius.juhyun.chung@emory.edu; 2 Department of Biostatistics and Bioinformatics, Rollins School of Public Health, Emory University, Atlanta, GA 30322, USA; ma3tian1wen2@emory.edu; 3 Department of Radiology and Imaging Sciences, Emory University, Atlanta, GA 30307, USA; phaethon.philbrook@emory.edu (P.P.); adam.ezra.goldman-yassen@emory.edu (A.E.G.-Y.); 4 Emory College of Arts and Sciences, Emory University, Atlanta, GA 30322, USA; toby.zhou@emory.edu; 5 Winship Cancer Institute, Emory University School of Medicine, Atlanta, GA 30322, USA

**Keywords:** brain tumor grading, amide proton transfer, chemical exchange saturation transfer

## Abstract

Background/Objectives: Accurate grading of brain gliomas is important, and amide proton transfer-weighted (APTw) MRI shows promise for non-invasive tumor differentiation. This study aimed to perform a comprehensive review and meta-analyses to demonstrate heterogeneity in both the diagnostic accuracy and quantitative consistency of APTw MRI in distinguishing high-grade gliomas (HGGs) from low-grade gliomas (LGGs), highlight issues with reporting standards and identify sources of heterogeneity through meta-regression. Methods: A systematic literature search was conducted between 1 January 2013 and 18 January 2026, following PRISMA guidelines. Peer-reviewed articles in English reporting diagnostic accuracy/contrast values of APTw MRI and study parameters were included. Principal component analysis (PCA) was used to extract the principal components (PCs) of the chemical exchange saturation transfer (CEST) contrast mechanism. Random-effects meta-analyses and univariate meta-regression models using individual CEST parameters and three PCs were performed. Forest plots with pooled estimates were generated. Leave-one-out meta-analysis (LOOMA) and complete case analysis were performed to examine the effects of outliers and missing data, respectively. Results: A total of 31 studies were included. Meta-analyses of the AUC and mean difference demonstrated significant heterogeneity across the studies (I^2^ = 73.9% & 78.2%, *p* < 0.001). The mean difference was moderated by one SD within the mean of the readout PC (*p* = 0.034) and the total PC (*p* = 0.02). The heterogeneity for the AUC and group mean difference was not substantially reduced by moderating nor LOOMA. The results of the meta-regression using all the data were similar to those using only data with no missing parameters. Conclusions: While APTw MRI shows promise for non-invasively distinguishing glioma grades, substantial heterogeneity in the study parameters limits generalizability. To improve consistency and comparability across studies, full reports of imaging parameters and standardization of APTw protocols are essential.

## Simple Summary

Significantly large heterogeneity in diagnostic accuracy measured by area under the curve (AUC) from receiver operating characteristic analysis and quantitative consistency measured by mean difference in amide proton transfer-weighted (APTw) MRI was observed and partially attributable to scan parameters, highlighting the need for full protocol reporting and standardization. Heterogeneity in mean difference was moderated by the total parameter set. Meta-regression suggests that the current set of imaging parameters could improve from further optimization. Moreover, outlier analysis suggests that parameterization of APTw MRI has yet to reach consensus.

## 1. Introduction

Magnetic Resonance (MR) imaging plays an increasingly vital role in the diagnosis, characterization, and monitoring of brain tumors [1,2,3], with over 80,000 new cases being discovered every year [4]. A key driver for the continued expansion of MR imaging applications is its distinct tissue contrast that differentiates tumors from surrounding tissue [5]. The World Health Organization has established a glioma grading system based on histopathological and molecular features [6], categorizing tumors from low-grade (I/II) to high-grade (III/IV) [7]. Accurate tumor grading plays a critical role in prognosis estimation and treatment planning, directly impacting patient outcomes and quality of life.

Routine clinical brain tumor protocols often include structural, contrast-enhanced, diffusion [8] and perfusion scans [1,9], and even magnetic resonance spectroscopy [1,10,11]. However, studies have demonstrated the need for expanding the tools available for tumor imaging [12,13]. Over the last 20 years, chemical exchange saturation transfer (CEST) MRI has emerged as a sensitive approach to map mobile proteins/peptides and microenvironmental pH [14,15,16]. Amide proton transfer-weighted (APTw) MRI has been the most studied, which targets amide protons that are ubiquitous in the primary structure of mobile endogenous peptides and proteins. Studies have shown that APTw MRI is potentially more effective in grading these tumors than conventional imaging [17], as well as diffusion and perfusion MRI [18,19]. It has also shown promise in predicting tissue response to treatment, differentiating between tumor progression and treatment response [20,21], and identifying IDH mutation status [22,23,24,25], 1p/19q codeletion [26], and MGMT [24,27]. However, while the diagnostic potential of APTw imaging has been extensively studied, routine analysis often does not account for differences that govern the source of APTw contrast. Compared to other modalities, altering imaging parameters could substantially impact CEST contrast to the point where additional sources of CEST effects could potentially dilute the observed APTw contrast. Nevertheless, the potential impact of such scan parameters could be hard to assess if they are not fully reported, resulting in a difference in the cutoff values used for diagnostics, impairing quantification consistency across studies.

The diagnostic potential of CEST MRI arises from the interplay of several intrinsic and extrinsic parameters. To generate CEST contrast, a radiofrequency pulse is applied at a frequency resonant with a targeted labile proton group, and saturation is then transferred to the bulk tissue water pool during the Radio Frequency (RF) saturation, conferring the CEST contrast. As a result, the dilute labile proton signal and exchange properties can be amplified and inferred from the abundant water signal. For APTw imaging, beyond the chemical shift of the amide protons (approximately 3.5 ppm), two key intrinsic properties govern APT contrast: (1) the concentration of exchangeable amide protons, potentially reflective of increased protein synthesis during cell proliferation, and (2) the exchange rate of these protons with water, which is pH-dependent and may be modulated by metabolic changes, such as the Warburg effect. However, the complex interactions among these parameters and how they are influenced by the specifics of the MR pulse sequence are still under continuous study. This complexity has led to variation in APTw imaging protocols and processing across studies, particularly regarding the parameters of the CEST sequence. A summary of these parameters are described in Table 1.

CEST parameters can be broadly grouped into the following two factors. The first one is saturation: (a) average CEST RF B_1_ amplitude (B_1_ average, B_1,avg_) represents the average RF power applied during saturation. It modulates exchange rate sensitivity [15,28] and is distinct from peak B_1_, being related by duty cycle and pulse shape [29]. (b) CEST RF duty cycle (DC) defines the proportion of time during which saturation is applied relative to the total saturation duration (T_sat_). Reduced duty cycles can suppress sensitivity to slow chemical exchanges compared to high duty cycles (e.g., continuous wave saturation) [30,31,32]. (c) CEST RF saturation time (T_sat_) determines the degree to which the system approaches steady state under saturation. Fast-exchanging protons may exhibit maximal sensitivity in shorter durations, while slower exchanges exhibit maximal sensitivity in longer duration [28,33]. The second factor is the image readout: (a) Repetition Time (TR) is the time between successive saturation-readout cycles. TR influences relaxation recovery and, consequently, the reproducibility of the steady-state signal [34]. (b) The image readout type encompasses sequence characteristics, such as dimensionality (2D vs. 3D), echo type (gradient-echo vs. spin-echo), and vendor-specific implementations that can impact image timing and sensitivity. Exchange Rate Tuning parameters that affect the CEST saturation, together with CEST RF saturation frequency, determine which labile protons are imaged using CEST MRI, whether they are amide protons or other overlapping moieties. Read-out parameters primarily determine signal sensitivity, as they are independent of the applied saturation. Among these two groups of parameters, parameters related to timing affect the approximation of the exchange system as existing in a steady state, which simplifies many properties in CEST, in a similar way to how assumptions of equilibrium simplify reaction kinetics in the chemistry field. Together, these groupings may represent how these parameters could be considered with respect to the CEST mechanism.

Given the variability in both intrinsic and extrinsic factors affecting APT signal generation, there has been a persistent effort in standardizing and further optimizing scan parameters for APTw MRI, including a recent white paper in 2022 [35]. Nevertheless, there continue to be substantial differences in reported APTw MRI protocols across centers, raising questions about the reproducibility and generalizability of APT-derived metrics for brain tumor grading. Some of the reported studies did not fully report the scan parameters needed, as described above, to properly evaluate their imaging protocols for APTw MRI. While there have been meta-analyses that have studied the diagnostic accuracy of APT imaging by aggregating Area Under the Curve (AUC) values from Receiver Operating Characteristic (ROC) analyses, APTw MRI is semi-quantitative, and the reliability of the quantitative differences between glioma types must also be explored. Furthermore, while other differentiation diagnostics may hold additional clinical relevance, such as the differentiation of tumor recurrence from radiation necrosis, the volume of data regarding the differentiation between HGG and LGG is still the largest available for an in-depth analysis of imaging parameter relevance.

In this meta-analysis, we systematically identified and analyzed 31 studies [10,11,18,19,22,23,24,25,26,36,37,38,39,40,41,42,43,44,45,46,47,48,49,50,51,52,53,54,55,56,57] that reported the use of APTw imaging for glioma grading and evaluated heterogeneity in both the diagnostic accuracy of APT imaging and the mean difference in contrast values between high-grade and low-grade gliomas. We observed the impact of incomplete parameter reporting and attempted to use principal component analysis to interpret decision-making behavior for parameter settings across various parameters with respect to their role in APTw imaging. Additionally, we explored whether the observed heterogeneity could be explained by variations in CEST imaging parameters using meta-regression. In addition, we quantify the results of our investigation by exploring two additional questions: 1. Using the first principal component of each set of parameters as a surrogate for similarity across parameters, does the average choice of parameters (or closest to the mean of the principal component) represent more optimized diagnostic accuracy or larger mean difference versus outliers (or S.D. outside of the mean)? 2. Using metrics such as leave-one-out meta-analysis (LOOMA), are there outliers for our meta-analysis of diagnostic accuracy (AUC) and quantitative consistency (M.D.), even with recent efforts to push for standardization of parameters and quantification?

## 2. Materials and Methods

### 2.1. The Search Strategy

This systematic review and meta-analysis were conducted in accordance with the Preferred Reporting Items for Systematic Reviews and Meta-Analyses (PRISMA) guidelines [58]. A comprehensive computerized search of the PubMed, Medline, and Embase databases was performed to identify relevant studies published between 1 January 2013 and 18 January 2026 (described in the Supplementary Materials). The search aimed to assess the diagnostic performance and consistency of APTw MRI in glioma grading. The search terms were structured to capture the following three domains: (1) APT imaging, (2) brain tumor patients, and (3) glioma grading. These concepts were combined with Boolean operators and synonyms, as detailed in the Supplementary Materials. This study was conducted in strict accordance with a pre-defined internal protocol, which was registered retrospectively in the INPLASY register as INPLASY202640073 (10.37766/inplasy2026.4.0073). To ensure transparency and reproducibility, the research follows the PRISMA 2020 (Preferred Reporting Items for Systematic Reviews and Meta-Analyses) guidelines. All the methods, inclusion criteria, and analysis plans were established prior to the data extraction phase to minimize bias.

### 2.2. Inclusion Criteria

In order to build on previous analyses of diagnostic accuracy, eligible studies met the following criteria: published in English in a peer-reviewed journal; included patients with histopathologically confirmed gliomas, where biopsy served as the reference standard for tumor grading; employed pre-treatment APT imaging as the index test for glioma grading; and reported or provided sufficient data to derive ROC metrics, like sensitivity and specificity, but specifically AUC. They were subsequently analyzed to examine sufficient reporting standards to evaluate the quantitative consistency of the reported metrics. The exclusion criteria included the following: animal or laboratory studies, reviews, meta-analyses, case reports, conference abstracts or presentations, and studies lacking either histopathological confirmation or usable APT grading data.

### 2.3. Data Extraction

The data were extracted and organized using Microsoft Excel. The extracted variables included the following: study identifiers (author, year, sample size); group-specific APTw mean differences, standard deviations, and sample sizes; reported AUC values, including detailed CEST sequence parameters (magnetic field strength (B_0_), RF saturation amplitude (B_1_) and duration (T_sat_), repetition time (TR)); image readout (e.g., spin-echo vs. gradient-echo, 2D vs. 3D); cutoff thresholds for classification; and three principal component (PC) scores derived from saturation preparation, magnetization recovery, and steady-state domains (see below). Quality assessment of the selected studies was performed using the Quality Assessment of Diagnostic Accuracy Studies-2 (QUADAS-2; Supplementary Figure S1) [59].

### 2.4. Principal Component Analysis of Grouped Parameters

To investigate how technical parameters may influence APT contrast and diagnostic performance, CEST-related variables were grouped based on their role in the APT contrast mechanism: exchange rate tuning, read-out, and steady-state behavior. Each group of parameters were normalized by their mean and standard deviation and then underwent principal component analysis (PCA) to reduce dimensionality and extract eigenvectors representing the largest coherent variance across parameters in that group, with each additional sequential orthogonal eigenvector ordered down to the smallest variance. The first principal component from each group (i.e., the component with the highest eigenvalue) was retained for subsequent meta-regression. Specifically, we have the exchange PC, the readout PC, and the steady-state PC, whose groupings are derived conceptually based on their effect on CEST imaging, as denoted in Table 1. Each principal component was treated as a covariate in subsequent univariate meta-regression analyses to assess its moderating effect on diagnostic accuracy and APT contrast.

### 2.5. Statistical Analysis

We analyzed two outcomes of interest: AUC and mean difference between two groups using random-effects meta-analyses. For the AUC, the variance estimate of AUC was computed by the derivation set forward by Hanley et al. [60]. Let *θ* denote AUC, we have $Var\left(\theta \right)=\frac{\theta \left(1\;-\;\theta \right)\;+\;\left(n_{1}\;-\;1\right)\left(Q_{1}\;-\;\theta^{2}\right)\;+\;\left(n_{2}\;-\;1\right)\left(Q_{2}\;-\;\theta^{2}\right)}{n_{1}n_{2}},$ where $Q_{1}=\frac{\theta }{2\;-\;\theta },\;Q_{2}=\frac{2\theta^{2}}{1\;+\;\theta }$, and $n_{1},\;n_{2}$ are sample sizes for groups 1 and 2, respectively. Since the AUC value ranged from 0 to 1 and most of the reported AUC values were not on the boundaries, a logit transformation was applied to the raw estimates, while the delta method was used to compute the standard error (SE) of logit(AUC). The random-effects meta-analyses were performed on the transformed scale, and the results were transformed back to the original range between 0 and 1. The 95% confidence intervals of the AUC values for each study may be asymmetric due to the non-linear transformation. For the mean differences, the variance estimate of the mean difference was computed by pooled variance assuming unequal variance between the two groups. Let $\mu_{1},$ $\mu_{2}$, and δ be the sample mean intensity for group 1, the sample mean intensity for group 2, and the mean difference, respectively. We have $Var\left(\delta \right)=\frac{v_{1}}{n_{1}}+\frac{v_{2}}{n_{2}}$, where $v_{1}$ and $v_{2}$ are the sample variances for groups 1 and 2, respectively. No transformation was applied to the mean difference estimates for meta-analysis.

For both meta-analyses, forest plots with pooled estimates, 95% confidence intervals (CIs), and relative weights were presented. The $I^{2}$ and $\tau^{2}$ statistics and *p*-values from the Cochran’s Q-test for residual heterogeneity were reported to quantify the heterogeneity of the selected studies [61,62]. Funnel plots and *p*-values from Egger’s tests were presented to examine the publication bias [63]. Meta-regression on selected CEST parameters and PCs was performed to identify the potential source of heterogeneity. To examine the study parameter specific outlier effect (parameter consensus), a binary variable indicating whether the PC value fell within its mean by 1 SD was created, denoted as PC within 1 SD. Studies with unknown CEST parameters were removed for meta-regression. To address the heterogeneity introduced by changes in glioma classification over time, an indicator of publication time was included. The indicator had three categories: 2007–2016, 2017–2021, and 2022-present. The meta regression analyses were fitted with one CEST parameter each time for feature screening. The *p*-values from the omnibus test for moderators and $I^{2}$ and $\tau^{2}$ statistics were reported to quantify the statistical significance of certain CEST parameters on the variability of the AUC values and mean differences across the studies.

Since data imputation is usually performed on the subject level within each study conditional on observed covariates of other subjects, it is not applicable to our case, as missing parameters are study-specific. To address the issue with varying levels of missing study parameters, we performed a sensitivity check on meta-analysis and meta-regression using a more restrictive complete-case only analysis in addition to the liberal case, which maximizes the sample size by including studies that may have missing parameters, provided those parameters do not preclude the analysis itself. For the restrictive case, we excluded any studies with incomplete parameters of interest. In this case, the number of articles was consistent with all the moderator analyses. To make the results convenient to read, we show the results under two conditions in parallel and specifically marked the number of missing studies. Finally, since missing covariates did not affect the forest plot nor the funnel plot, we did not include those plots from the complete case analysis.

To examine the outlier issue, we adopted the conventional approach: leave-one-out meta-analysis (LOOMA). We reran the meta-analysis by excluding each study. The updated I^2^ and tau^2^ statistics and *p*-values from the Cochran’s Q-test for residual heterogeneity were reported. In addition, we produced a Baujat plot to identify studies that contributed most to heterogeneity with a high influence on the overall pooled measure. Studies with high-impact outliers appear on the top-right corner of the plot.

All the statistical analyses were performed in R Version 4.3.1 (Vienna, Austria) [64] with “metafor” package [65]. *p*-values smaller than 0.05 were considered statistically significant.

## 3. Results

**Figure 1:**
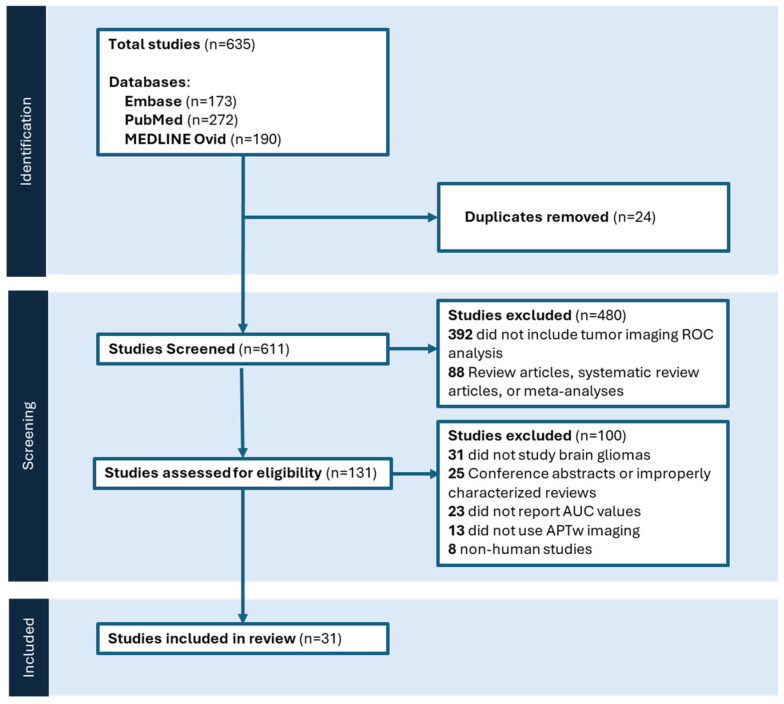
Prisma flowchart detailing the literature search and screening process.

Of the 635 studies initially reviewed, a total of 31 studies met the inclusion criteria and were incorporated into the meta-analysis (Figure 1). The study-level details—including AUC values, mean differences, comparison groups, sample sizes, and reported CEST parameters—are summarized in Table 2 and Table 3.

The principal components derived from grouping the CEST parameters are presented in Supplementary Tables S1–S4, along with a description of the corresponding eigenvectors. The variance across the exchange PC comprised 40%, which was only slightly more than in its orthogonal directions (32% and 29%), meaning there was not a strong consensus in the differences of these parameters across all the studies. Similarly, for a 1 S.D, movement along the exchange PC, 41% is along T_sat_ and 38% is increasing B_1,sat_, while 21% is decreasing DC. For the readout PC, nearly 66% of the variance was along the first two orthogonal directions (34% and 31%) out of 7 eigenvectors. Along the first PC for a 1 S.D, movement, 37% is along TR, and 36% is increasing T_rec_, underlying the importance of timing parameters. For the steady state PC, 67% of the variance is along TR and T_sat_ moving equally in the same direction. Across the entire parameter set, over half of the variance is explained by the first two eigenvectors (28% and 25%). For the first total PC, for a 1 S.D. change, 20% is along B_1_, with 14% and 12% being along timing parameters: T_sat_ and TR in the same direction.

### 3.1. Covariate-Free Meta-Analysis

All 31 studies reported AUC values for distinguishing LGG from HGG using APTw MRI, while only 24 studies reported the mean difference and SE in contrast values between the LGG and HGG groups.

#### 3.1.1. AUC

**Figure 2:**
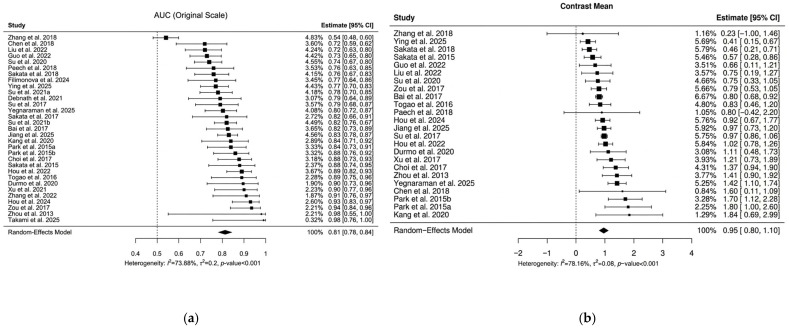
Forest plots of AUC (**a**) and Difference of Mean Contrast (**b**) comparing LGG to HGG among 31 studies [10,11,18,19,22,23,24,25,26,36,37,38,39,40,41,42,43,44,45,46,47,48,49,50,51,52,53,54,55,56,57]. Seven studies were excluded in (**b**) due to unknown mean or SE values. The I^2^ heterogeneity measure, τ^2^ (absolute measure of variance of the true effect sizes across studies), and *p*-value for Cochran’s Q test for residual heterogeneity were 73.9%, 0.2, and <0.001 for AUC, respectively and for the Mean Difference in Contrast they were 78.2%, 0.08, and <0.001, respectively.

**Figure 3:**
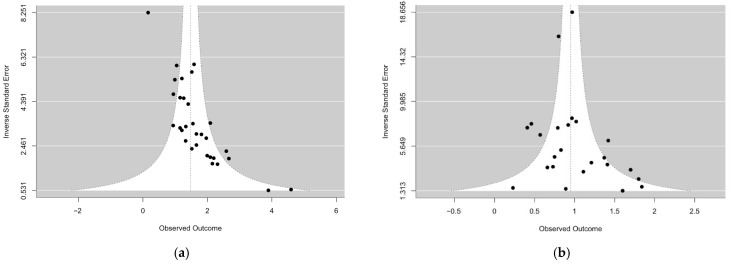
Funnel plots of AUC values (**a**) and Difference of Mean Differences (**b**) comparing LGG to HGG.

The meta-analysis yielded a pooled AUC of 0.81 (95% CI: [0.78, 0.84]; Figure 2a). However, the AUC values ranged from 0.54 to 0.99. Significantly high heterogeneity was detected (I^2^ = 73.9%, τ^2^ = 0.2, *p* < 0.001). The funnel plot did indicate some potential publication bias, with 7 out of 31 studies (22.6%) falling outside the expected confidence limits (Figure 3a), and the Egger’s test confirmed this asymmetry (*p* < 0.001). The restrictive complete-case analysis showed similar heterogeneity: Among 19 studies, the I^2^ and τ^2^ values and *p*-value from residual heterogeneity were 83.0%, 0.33, and <0.001, respectively. Analysis of logit(AUC) is additionally presented in the Supplementary Materials (Figure S2).

#### 3.1.2. Mean Difference

The pooled mean difference in contrast between the groups from the meta-analysis was 0.95 (95% CI: [0.80, 1.10]; Figure 2b). However, the reported values ranged from 0.23 to 1.84. Significantly high heterogeneity was detected with statistical significance (I^2^ = 78.2%, τ^2^ = 0.08, *p* < 0.001). The funnel plot for the mean difference in contrast between the groups also showed slight asymmetry, with 7 out of 24 studies (29.2%) outside the funnel bounds (Figure 3b), and Egger’s test returned a *p*-value of 0.06. However, a lack of asymmetry may not suggest a lack of publication bias. The restrictive complete case analysis showed similar heterogeneity: Among 18 studies, the I^2^ and τ^2^ values and *p*-value from residual heterogeneity were 75.5%, 0.084, and < 0.001, respectively.

### 3.2. Univariate Meta-Regression

To analyze potential moderators for heterogeneity, univariate meta-regression was performed on both AUC and the Mean Difference in Contrast between the groups. Moderators include each parameter, PCs from the parameter set, 1 SD outside each PC for consensus evaluation, and a time indicator to reflect the protocol change over time.

#### 3.2.1. AUC

For the maximum sample scenario, none of the study parameters, PCs or their binary status within 1 SD, nor the time indicator showed statistical significance in moderating tests. The means of LGG and HGG and the group mean differences and cutoff values, despite significant moderating effects, were not included for meta-regression because they were not study parameters. With respect to parameter consensus, there was no demonstrable statistical significance between within and outside 1 SD of the mean for any of the first principal components of the parameter groups used for PCA, including the total set of parameters. The meta-regression results for the complete case analysis were similar with the only exception for saturation time. It reduced the I^2^ from 83.0% to 79.2% and accounted for 4.8% of the variability (*p* = 0.04 for the moderating effect). Detailed information can be found in Table 4.

#### 3.2.2. Mean Difference

The total PC showed a significant moderating effect that reduced the total heterogeneity from 75.5% to 67.1% and accounted for 23.5% of the heterogeneity (*p* = 0.02). Despite the lack of statistical significance of read-out PC and steady-state PC, the consensus measure of read-out PC reduced the total heterogeneity from 75.5% to 68.9% and accounted for 23.1% of the variability (*p* = 0.034). In addition, the consensus measure of steady-state PC and total PC also reduced the total heterogeneity from 75.5% to 71.4% and 71.1% and accounted for 12.9% and 0.6% of the variability, respectively. The *p*-values for the omnibus moderator tests were both below 0.1. The meta-regression results by complete case analysis were also similar, except that the scanning dimensionality (3D vs. 2D) significantly reduced the heterogeneity from 75.5% to 62.4% and accounted for 39.0% of the variability (*p* = 0.031).

In this case, the average parameters currently utilized for read-out among the studies may not be optimized to provide maximal mean difference in APTw contrast between glioma groups. Detailed information can be found in Table 5.

### 3.3. Outlier Evaluation Using Baujat Plots and LOOMA

**Figure 4:**
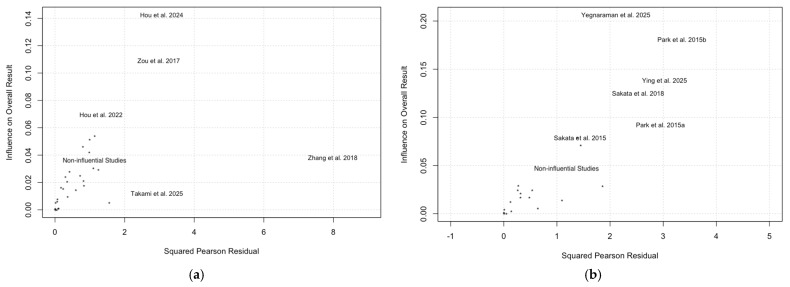
Baujat plots to visualize the heterogeneity of each study on AUC [10,11,18,19,22,23,24,25,26,36,37,38,39,40,41,42,43,44,45,46,47,48,49,50,51,52,53,54,55,56,57]. (**a**) and mean difference in contrast to comparing LGG to HGG (**b**). Hou et al. [23] 2024, Zou et al. [53] 2017, and Zhang et al. [51] 2018 were the main studies that had large Pearson residuals with large influence on the pooled AUC values, while Yegnaraman et al. [55] 2025, Park et al. [44] 2015b, Ying et al. [54] 2025, and Sakata et al. [46] 2018 were the main studies that had large Pearson residuals with large influence on the pooled mean difference of contrast.

To examine the stability of the meta-analysis across the studies, outlier analysis was performed using leave-one-out meta-analysis (LOOMA; Table 6 and Table 7) and Baujat plots (Figure 4).

#### 3.3.1. AUC

LOOMA revealed that omission of Zhang et al. [51] 2018 resulted in a 30.9% reduction in I^2^ and a 60% reduction in τ^2^ (Table 5). Looking at the Baujat plots for AUC (Figure 4a), the main studies with the largest Pearson residuals with large influence on the pooled AUC values were Hou et al. [23] 2024, Zou et al. [53] 2017, and Zhang et al. [51] 2018. Although the field had developed as of 2024, some outlying studies were still forthcoming. The omission of each study did not substantially resolve the heterogeneity issue, as an I^2^ of 52% was still considered moderate.

#### 3.3.2. Mean Difference in Contrast Comparing LGG to HGG

LOOMA revealed that omission of Ying et al. [54] 2025 and Yegnaraman et al. resulted in a 5.55/4.34% reduction in I^2^ and a 25/25% reduction in τ^2^, respectively (Table 6). Yegnaraman et al. [55] 2025, Park et al. [44] 2015b, Ying et al. [54] 2025, and Sakata et al. [46] 2018 were the main studies that had large Pearson residuals with large influence on the pooled mean difference in contrast (Figure 4b). The I^2^ values after LOOMA on the mean difference in contrast comparing LGG to HGG were still larger than 70%, indicating high heterogeneity. In addition, the most significant outliers in LOOMA that were also identified by the Baujat plots were both published in 2025, suggesting that the field of study has not come to a stable consensus yet for the mean difference in APTw contrast between glioma groups.

## 4. Discussion

Our study conducted a systematic literature review and meta-analyses of 31 studies evaluating the diagnostic utility and quantitative consistency of APT imaging for distinguishing HGG from LGG to highlight the large heterogeneity among existing studies and the need for standardized protocols and reporting of parameters and results. While previous meta-analyses [66,67,68] have been performed to study diagnostic performance through AUC values, due to the importance of communicability between groups, we have extended the analysis to the consistency of the quantitative mean difference in measured contrast between groups. As CEST sensitivity is essentially a semi-quantitative value, the impact of study parameters on the heterogeneity of the gross difference measured in contrast between groups is crucial to its communicative value between study groups. We performed both meta-analyses and univariate meta-regression to explore the influence of individual imaging parameters, principal component transformations of the total parameter set, three sub-groups of parameters, and a time indicator on the observed outcomes.

In concurrence with previous studies, we found, along with the mean difference in contrast (I^2^ > 75%), the AUC exhibited significant heterogeneity (I^2^ > 70%). The I^2^ values after LOOMA for AUC and mean difference were still moderate to large, indicating that the omission of studies did not substantially resolve the heterogeneity issue and motivated us to find potential moderators to account for the large heterogeneity. While Suh et al. [67] identified B_1_ as a significant moderator for AUC heterogeneity, with the addition of recent studies, neither B_1_ nor any other single parameter has a significant effect in moderating AUC heterogeneity when using a liberal approach using all study data available. We added a sensitivity check running the regression with a restricted set of studies reduced to only studies that reported all parameters needed to validate their APTw protocol. In contrast, when using a more restrictive approach, saturation time is a significant moderator for heterogeneity. To understand why the smaller set that properly reported parameters may exhibit more specific significance, we attempted to analyze the behavior in the parameter setting using PCA. We found that while parameters related to read-out or steady state did exhibit some coherence, especially with respect to timing parameters, parameters related to exchange rate were modulated with significantly less coherence. This lack of parameter coherence with respect to mechanism may have muddied any correlation with individual parameters in attempting to establish linkages through univariate regression.

Like the liberal regression for AUC, there were no regressors among the individual parameters for heterogeneity in the mean difference in contrast between the groups; however, analyzing the grouped parameters with principal component analysis revealed moderators in the primary principal components across all the parameters and near significance for parameters moderating the exchange rate tuning of APTw contrast. On the other hand, when running a restricted analysis, the dimensionality of the read-out became a significant moderator of heterogeneity in the mean difference between the groups along with the total PC.

These findings reinforce the need to standardize imaging protocols and, at the very least, the need to consistently report all imaging parameters. Our analysis comparing within a standardization of the mean of the first principal component of sets of parameters has demonstrated that there is yet to be a set of parameters that proves statistically optimal over outliers for diagnostic accuracy or quantitative consistency. In fact, analysis of the readout PC suggests that the current average parameters for readout may be suboptimal in maximizing the mean difference in contrast. Furthermore, the yet persisting report of outlying studies suggests that the field could benefit from further consensus.

One drawback in our study is the change in grading standards for high- and low-grade gliomas with the release of new WHO guidelines in 2007, 2016, and 2021. These guideline changes mark technological upgrades in classification from pure histology to the use of molecular markers to full molecular integration, which may impact grading standards. Unfortunately, using only the data reported in these papers, exact harmonization is untenable. Instead, we ran a sensitivity analysis using a time indicator to test the significance of these landmark changes and were unable to determine any statistical significance for time periods across these guideline changes.

Another key weakness in our analysis is that the volume of studies gathered for analysis was undermined by insufficient consistency in reporting standards for crucial parameters that are vital for understanding CEST behavior. Out of the 31 studies included, only 19 studies reported the minimum parameters required to qualify their APTw imaging protocol. For CEST, a change in imaging parameters can substantially change the imaging modality and the imaged moiety. Efforts to improve inter-study comparability should include broader adoption of standardized guidelines, such as those proposed in the clinical APT consensus paper [35], as well as efforts to harmonize study parameters once optimization is achieved. In addition to standardized protocols, the implementation of quantitative standardization techniques [54,69,70] may help minimize variability introduced by sequence parameters by harmonizing the influence of these parameters on output maps or enabling the calculation of parametric maps with more direct physiological meaning [71,72]. Moreover, routine reporting of the saturation module and readout parameters, especially those influencing exchange rate sensitivity, is necessary. Together, these initiatives can reduce the interpretive ambiguity caused by parameter heterogeneity and promote the reliable use of APT imaging as a biomarker in glioma diagnostics.

## 5. Conclusions

While APTw MRI shows promise for non-invasively distinguishing glioma grades, substantial heterogeneity in study parameters limits its generalizability. Significant heterogeneity was demonstrated in both diagnostic accuracy and quantitative consistency. While individual parameters alone were insufficient to moderate heterogeneity, PCA has revealed that the total parameter set does, in fact, moderate the heterogeneity of the quantitative difference between glioma groups. Meta-regression suggests the current set of imaging parameters could still benefit from further optimization, and the existence of recent outliers suggests that standardization of APTw MRI has yet to reach consensus. To improve consistency and comparability across studies, full reports of imaging parameters and standardization of APTw protocols are essential.

## Figures and Tables

**Table 1:** Description of parameters affecting CEST MRI acquisition.

Parameter		Effect	Description
Saturation	B_1_ average	Exchange Rate Tuning	Average RF power during saturation increases sensitivity; increased B_1,avg_ increases optimal exchange rate [15,28]
	Duty Cycle (DC)	Exchange Rate Tuning	Proportion of time during which saturation is applied relative to the total saturation duration; reduced DC can suppress slow CEST compared to high DC [29,30,31,32]
	CEST RF Saturation Time	Exchange Rate Tuning/Steady-State	Total Saturation Duration; fast-exchanging protons maximize sensitivity at shorter T_sat_, while slower exchanges maximize sensitivity closer to steady state [28,33]
Image Readout	Repetition Time	Image Readout	Time between successive saturation–readout cycles; relaxation recovery and reproducibility of steady-state signal [34]
	Dimension (2D/3D)	Image Readout	Sequence dimensionality affects timing, resolution and Signal-to-Noise Ratio (SNR)
	Echo (Gradient/Spin)	Image Readout	Type of sequence echo can affect sensitivity or timing
	Vendor (Scanner)	Image Readout	Scanner vendor affects access to pre-installed sequences

**Table 2:** Characteristics of glioma grading using APTw MRI in selected studies.

Authors	AUC	Cut-Off	HGG			LGG			Mean Diff.
			n	Mean	SD	n	Mean	SD	
1. Zhou et al. [52] 2013	0.98	1.92	8	2.5	0.55	6	1.09	0.42	1.41
2. Park et al. [44] 2015b	0.86	3.7	26	4	1.2	19	2.3	0.8	1.7
3. Park et al. [10] 2015a	0.84	1.72	30	2.9	1.6	10	1.1	0.9	1.8
4. Sakata et al. [45] 2015	0.88	1.21	18	1.35	0.44	8	0.78	0.3	0.57
5. Togao et al. [19] 2016	0.89	2.56	14	2.7	0.58	20	1.87	0.49	0.83
6. Bai et al. [36] 2017	0.83	NaN	26	2.1	0.2	18	1.3	0.2	0.8
7. Choi et al. [18] 2017	0.88	1.53	31	2.21	0.88	15	0.84	0.6	1.37
8. Sakata et al. [11] 2017	0.82	2.72	11	NaN	NaN	10	NaN	NaN	NaN
9. Su et al. [49] 2017	0.79	2.93	14	3.61	0.155	28	2.64	0.18	0.97
10. Zou et al. [53] 2017	0.94	2.34	26	2.77	0.35	25	1.98	0.58	0.79
11. Chen et al. [37] 2018	0.72	NaN	13	4.5	2.3	7	2.9	1.1	1.6
12. Paech et al. [24] 2018	0.76	3.66	25	3.96	1.32	6	3.07	1.5	0.89
13. Sakata et al. [46] 2018	0.76	1.26	34	1.33	0.46	15	0.87	0.39	0.46
14. Zhang et al. [51] 2018	0.54	NaN	16	4.46	1.44	16	4.23	2.06	0.23
15. Durmo et al. [39] 2020	0.90	2.38	13	2.6	0.97	9	1.49	0.5	1.11
16. Kang et al. [41] 2020	0.84	2.53	18	3.9	1.2	9	2.06	1.55	1.84
17. Su et al. [47] 2020	0.74	2.14	39	3.02	0.95	30	2.27	0.85	0.75
18. Debnath et al. [38] 2021	0.79	3.63	10	NaN	NaN	14	NaN	NaN	NaN
19. Su et al. [48] 2021a	0.78	NaN	25	NaN	NaN	39	NaN	NaN	NaN
20. Su et al. [26] 2021b	0.82	NaN	68	NaN	NaN	45	NaN	NaN	NaN
21. Xu et al. [25] 2021	0.9	1.55	17	2.25	0.86	13	1.04	0.48	1.21
22. Guo et al. [22] 2022	0.73	NaN	48	2.81	1.25	14	2.15	0.8	0.66
23. Hou et al. [41] 2022	0.89	2.72	48	3.27	0.65	33	2.25	0.47	1.02
24. Liu et al. [43] 2022	0.72	NaN	23	2.86	0.72	15	2.13	0.89	0.73
25. Zhang et al. [50] 2022	0.91	3.52	15	NaN	NaN	10	NaN	NaN	NaN
26. Filimonova et al. [40] 2024	0.77	NaN	37	NaN	NaN	5	NaN	NaN	NaN
27. Hou et al. [23] 2024	0.93	2.38	34	2.76	0.51	24	1.84	0.47	0.92
28. Ying et al. [54] 2025	0.77	NaN	49	1.87	0.62	23	1.46	0.49	0.41
29. Yegnaraman et al. [55] 2025	0.80	2.80	37	3.30	0.86	20	1.88	0.35	1.42
30. Takami et al. [56] 2025	0.99	NaN	19	2.91	NaN	6	0.44	NaN	2.47
31. Jiang et al. [57] 2025	0.83	NaN	83	2.90	0.82	54	1.93	0.58	0.97

**Table 3:** Imaging parameters for APTw MRI used in selected studies.

Authors	Readout Parameters						CEST Parameters			
	Scanner	B_0_	dim.	Protocol	Echo	TR	B_1_	T_sat_	DC	N_p_
1. Zhou et al. [52] 2013	Philips	3	3D	GRASE	Both	3	0.5	0.83	0.96	4
2. Park et al. [44] 2015b	Philips	3	3D	GRE	GRE	0.14	0.42	0.07	1	1
3. Park et al. [10] 2015a	Philips	3	3D	GRE	GRE	0.14	0.42	0.07	1	1
4. Sakata et al. [45] 2015	Siemens	3	3D	GRE	GRE	NaN	2	0.6	0.5	3
5. Togao et al. [19] 2016	Philips	3	2D	FSE	SE	5	2	2	1	40
6. Bai et al. [36] 2017	GE	3	2D	GRE	GRE	3.2	2	1	0.5	5
7. Choi et al. [18] 2017	Philips	3	3D	EPI	SE	3	2	0.8	1	4
8. Sakata et al. [11] 2017	Toshiba	3	2D	FSE	SE	9	1	0.83	0.97	25
9. Su et al. [49] 2017	GE	3	NaN	NaN	NaN	3	2	0.4	1	1
10. Zou et al. [53] 2017	Philips	3	2D	TSE	SE	3	2	0.83	0.96	4
11. Chen et al. [37] 2018	Siemens	3	2D	GRE	GRE	1.34	1.6	0.5	1	5
12. Paech et al. [24] 2018	Siemens	7	2D	GRE	GRE	NaN	1	3.75	0.6	150
13. Sakata et al. [46] 2018	Siemens	3	2D	GRE	GRE	NaN	2	0.6	0.5	3
14. Zhang et al. [51] 2018	GE	3	2D	EPI	SE	3	2	0.4	1	1
15. Durmo et al. [39] 2020	Siemens	3	3D	GRE	GRE	NaN	2	0.77	0.67	5
16. Kang et al. [41] 2020	GE	3	2D	SE	SE	3	2	0.4	1	1
17. Su et al. [47] 2020	GE	3	3D	GRE	GRE	3	2	0.6	1	3
18. Debnath et al. [38] 2021	Philips	3	NaN	NaN	NaN	3	2	0.8	1	4
19. Su et al. [48] 2021a	GE	3	2D	GRE	GRE	3	2	1.6	1	4
20. Su et al. [26] 2021b	GE	3	2D	EPI	SE	6.5	2	2	1	1
21. Xu et al. [25] 2021	GE	3	2D	GRE	GRE	4	2	2	1	4
22. Guo et al. [22] 2022	Siemens	3	48	SPACE	SE	3	2.5	1	1	10
23. Hou et al. [41] 2022	Philips	3	3D	FSE	SE	5.93	2	2	1	1
24. Liu et al. [43] 2022	Philips	3	NaN	NaN	NaN	3	2	0.8	1	4
25. Zhang et al. [50] 2022	Philips	3	NaN	NaN	NaN	6.3	NaN	NaN	NaN	NaN
26. Filimonova et al. [40] 2024	Philips	3	3D	EPI	SE	5.93	NaN	NaN	NaN	NaN
27. Hou et al. [23] 2024	Philips	3	3D	TSE	SE	5.93	2	2	1	1
28. Ying et al. [54] 2025	United Imaging	3	2D	FSE	SE	2	0.75	1	1	1
29. Yegnaraman et al. [55] 2025	Philips	3	3D	TSE	SE	6.31	2	2	1	40
30. Takami et al. [56] 2025	GE	3	2D	FSE	SE	3.03	2	2	1	1
31. Jiang et al. [57] 2025	Siemens	3	3D	SPACE	SE	3	2.5	1.1	0.91	10

**Table 4:** Missing values, ***I***^**2**^, ***R***^**2**^, ***τ***^**2**^, and *p*-values of the omnibus test for examining univariate moderator effects of AUC comparing LGG vs. HGG.

Covariate Name	Missing Value	***I***^**2**^ (%)	***R***^**2**^ (%)	***τ*** ^**2**^	*p*-Value
Null *	0 (12)	73.9 (83.0)	/	0.202 (0.330)	/
B_0_ **	0 (12)	74.9 (83.0)	0.0 (/)	0.211 (0.330)	0.55 (/)
**Dim. [3D]**	3 (12)	73.9 (82.1)	1.8 (0.0)	0.208 (0.337)	0.33 (0.56)
**Echo [Spin Echo]**	4 (12)	76.8 (83.5)	0.0 (0.0)	0.236 (0.358)	0.75 (0.75)
**TR**	4 (12)	75.8 (82.9)	2.4 (0.7)	0.216 (0.327)	0.26 (0.27)
**B_1_**	2 (12)	75.7 (83.6)	0.0 (0.0)	0.218 (0.356)	0.81 (0.81)
**T_sat_**	2 (12)	74.2 (79.2)	2.5 (4.8)	0.202 (0.267)	0.27 (**0.04**)
**PC-related Meta-Regression Analyses**					
**Exchange Rate Tuning**	2 (12)	75.4 (83.2)	0.0 (0.0)	0.214 (0.341)	0.56 (0.43)
**Exchange Rate Tuning within 1 SD**	2 (12)	75.8 (83.6)	0.0 (0.0)	0.218 (0.357)	0.90 (0.93)
**Read-out**	9 (12)	80.2 (82.9)	0.1 (0.2)	0.263 (0.329)	0.32 (0.25)
**Read-out** **within 1 SD**	9 (12)	79.6 (82.1)	2.0 (4.6)	0.258 (0.314)	0.44 (0.34)
**Steady State**	6 (12)	76.1 (81.9)	7.3 (6.8)	0.212 (0.307)	0.14 (0.14)
**Steady State** **within 1 SD**	6 (12)	75.0 (82.1)	12.4 (4.6)	0.201 (0.314)	0.14 (0.34)
**Total**	9 (12)	80.6 (83.2)	0.0 (0.0)	0.272 (0.343)	0.55 (0.67)
**Total PC** **within 1 SD**	9 (12)	79.7 (83.0)	1.4 (1.0)	0.260 (0.326)	0.23 (0.23)
**Protocol Time Shift (2015, 2021)**	0 (12)	72.4 (82.6)	5.4 (0.0)	0.191 (0.342)	0.23 (0.50)

Liberal analysis (maximum studies) is expressed with restricted analysis (no missing values) in parentheses. * The first row corresponds to the original meta-analysis without covariate adjustment. ** For B_0_, the moderator effect was not available using complete case analysis because all the included studies used the same B_0_ value.

**Table 5:** Missing values, ***I***^**2**^, ***R***^**2**^, ***τ***^**2**^, and *p*-values of the omnibus test for examining univariate moderator effects of mean difference in contrast comparing LGG vs. HGG.

Covariate Name	Missing Value	***I***^**2**^ (%)	***R***^**2**^ (%)	***τ*** ^**2**^	*p*-Value
Null *	7 (13)	78.2 (75.5)	/	0.079 (0.084)	/
B_0_ **	7 (13)	79.2 (75.5)	0.0 (/)	0.080 (0.085)	0.93 (/)
**Dim. [3D]**	9 (13)	74.1 (62.4)	3.9 (39.0)	0.09 (0.052)	0.26 (**0.031**)
**Echo [Spin Echo]**	9 (13)	76.7 (73.3)	0.0 (0.0)	0.103 (0.093)	0.89 (0.25)
**TR**	11 (13)	78.9 (77.9)	0.0 (0.0)	0.079 (0.099)	0.70 (0.60)
**B_1_**	7 (13)	79.3 (77.2)	0.0 (0.0)	0.084 (0.094)	0.13 (0.20)
**T_sat_**	7 (13)	78.0 (77.3)	0.0 (0.0)	0.085 (0.097)	0.99 (0.51)
**PC-related Meta-Regression Analyses**					
**Exchange Rate Tuning**	7 (13)	75.6 (75.4)	1.3 (0.0)	0.078 (0.092)	0.066 (0.17)
**Exchange Rate Tuning within 1 SD**	7 (13)	77.4 (75.0)	0.0 (0.0)	0.089 (0.098)	0.51 (0.79)
**Read-out**	13 (13)	76.6 (76.6)	0.0 (0.0)	0.099	0.57 (0.57)
**Read-out** **within 1 SD**	13 (13)	68.9 (68.9)	23.1 (23.1)	0.065 (0.065)	0.034 **(0.034)**
**Steady State**	11 (13)	78.4 (77.7)	0.0 (0.0)	0.079 (0.099)	0.67 (0.56)
**Steady State within 1 SD**	11 (13)	72.5 (71.4)	15.3 (12.9)	0.057 (0.074)	0.062 (0.094)
**Total**	13 (13)	67.1 (67.1)	23.5 (23.5)	0.065 (0.065)	0.02 **(0.02)**
**Total PC within 1 SD**	13 (13)	71.1 (71.1)	0.6 (0.6)	0.084 (0.084)	0.096 (0.096)
**Protocol Time Shift (2015, 2021)**	7 (13)	79.6 (72.7)	0.0 (0.5)	0.092 (0.084)	0.50 (0.19)

Liberal analysis (maximum studies) is expressed with restricted analysis (no missing values) in parentheses. * The first row corresponds to the original meta-analysis without covariate adjustment. ** For B_0_, the moderator effect was not available using complete case analysis because all the included studies used the same B_0_ value.

**Table 6:** Summary table for leave-one-out meta-analysis (LOOMA) for AUC.

Removed Study	AUC	CI Low	CI Upper	Q-Value	*τ* ^2^	*I* ^2^
Zhang et al. [51] 2018	0.82	0.79	0.84	60.29	0.08	51.07
Chen et al. [37] 2018	0.82	0.78	0.85	147.98	0.21	74.37
Liu et al. [43] 2022	0.82	0.78	0.85	147.17	0.2	73.71
Guo et al. [22] 2022	0.82	0.78	0.85	147.34	0.21	73.57
Su et al. [47] 2020	0.82	0.78	0.85	147.78	0.21	73.46
Paech et al. [24] 2018	0.82	0.78	0.85	148.79	0.21	74.88
Sakata et al. [46] 2018	0.82	0.78	0.85	148.75	0.21	74.47
Filimonova et al. [40] 2024	0.82	0.78	0.85	148.83	0.21	74.97
Ying et al. [54] 2025	0.82	0.78	0.85	148.83	0.21	74.23
Su et al. [48] 2021a	0.82	0.78	0.85	148.76	0.21	74.66
Debnath et al. [38] 2021	0.82	0.78	0.84	148.73	0.21	75.07
Su et al. 2017	0.82	0.78	0.84	148.66	0.21	75.02
Yegnaraman et al. [55] 2025	0.82	0.78	0.84	148.11	0.21	74.82
Sakata et al. [11] 2017	0.81	0.78	0.84	148.31	0.21	75
Su et al. [26] 2021b	0.81	0.78	0.84	145.59	0.22	74.21
Bai et al. [36] 2017	0.81	0.78	0.84	147.43	0.21	74.9
Jiang et al. [57] 2025	0.81	0.78	0.84	143.31	0.21	73.93
Kang et al. [41] 2020	0.81	0.78	0.84	147.56	0.21	74.86
Park et al. 2015a	0.81	0.78	0.84	147.01	0.21	74.81
Park et al. [44] 2015b	0.81	0.78	0.84	145.55	0.21	74.46
Choi et al. [18] 2017	0.81	0.78	0.84	144.3	0.2	74.06
Sakata et al. [45] 2015	0.81	0.78	0.84	146.26	0.2	74.34
Hou et al. [41] 2022	0.81	0.78	0.84	139.34	0.19	73.03
Togao et al. [19] 2016	0.81	0.78	0.84	145.79	0.2	74.17
Durmo et al. [39] 2020	0.81	0.78	0.84	146.26	0.2	74.23
Xu et al. [25] 2021	0.81	0.78	0.84	145.17	0.2	73.95
Zhang et al. [50] 2022	0.81	0.78	0.84	145.42	0.2	73.96
Hou et al. [23] 2024	0.81	0.78	0.84	139.28	0.18	72.06
Zou et al. [53] 2017	0.81	0.78	0.84	141.01	0.18	72.55
Zhou et al. [52] 2013	0.81	0.78	0.84	146.79	0.2	74.35
Takami et al. [56] 2025	0.81	0.78	0.84	145.15	0.2	74.17

**Table 7:** Summary table for leave-one-out meta-analysis (LOOMA) for mean difference.

Removed Study	Mean Difference	CI Low	CI Upper	Q-Value	*τ* ^2^	*I* ^2^
Zhang et al. [51] 2018	0.96	0.81	1.11	74.24	0.08	79.05
Ying et al. [54] 2025	0.98	0.84	1.12	61.98	0.06	73.82
Sakata et al. [46] 2018	0.98	0.83	1.12	63.49	0.07	74.89
Sakata et al. [45] 2015	0.97	0.82	1.12	70.53	0.08	77.68
Guo et al. [22] 2022	0.96	0.81	1.11	74.66	0.08	79.41
Liu et al. [43] 2022	0.96	0.81	1.11	75	0.08	79.59
Su et al. [47] 2020	0.96	0.81	1.11	74.92	0.08	79.61
Zou et al. [53] 2017	0.96	0.81	1.12	74.77	0.09	79.33
Bai et al. [36] 2017	0.96	0.81	1.12	72.64	0.09	76
Togao et al. [19] 2016	0.96	0.81	1.11	75.24	0.09	79.78
Paech et al. [24] 2018	0.95	0.8	1.1	75.34	0.08	79.21
Hou et al. [23] 2024	0.96	0.8	1.11	75.28	0.09	79.55
Jiang et al. [57] 2025	0.95	0.8	1.11	74.89	0.09	79.36
Su et al. [49] 2017	0.95	0.8	1.11	72.16	0.09	75.43
Hou et al. [41] 2022	0.95	0.79	1.1	74.17	0.09	79.29
Durmo et al. [39] 2020	0.95	0.8	1.1	74.85	0.08	79.43
Xu et al. [25] 2021	0.94	0.79	1.09	73.63	0.08	78.99
Choi et al. [18] 2017	0.93	0.78	1.07	70.54	0.07	77.43
Zhou et al. [52] 2013	0.93	0.79	1.08	71.25	0.07	77.6
Yegnaraman et al. [55] 2025	0.92	0.78	1.06	64.19	0.06	74.77
Chen et al. [37] 2018	0.94	0.8	1.09	74.47	0.08	78.86
Park et al. 2015b	0.92	0.78	1.06	67.89	0.07	75.43
Park et al. [10] 2015a	0.93	0.79	1.07	70.33	0.07	77.01
Kang et al. [41] 2020	0.94	0.79	1.08	72.73	0.08	78.29

## Data Availability

Data generated by the authors or analyzed during this study were extracted from the studies analyzed and are available with their doi in the following references: References [10,11,18,19,22,23,24,25,26,36,37,38,39,40,41,42,43,44,45,46,47,48,49,50,51,52,53,54,55,56,57].

## Supplementary Materials

The following supporting information can be downloaded at: https://www.mdpi.com/article/10.3390/tomography12050065/s1. Supplementary Materials S1—Search List, Principal Component Analysis; Supplementary Materials S2—PRISMA Checklist [73]; Table S1: Exchange Principal Component: Exchange Rate Tuning; Table S2: Read-Out Principal Component: Magnetization Recovery; Table S3: Steady-state PC; Table S4: Total Principal Component: Total Parameters; Figure S1: QUADAS-2: Quality Assessment Results; Figure S2: Forest plot of logit(AUC) comparing LGG to HGG among 31 studies.

## Abbreviations

The following abbreviations are used in this manuscript:

APTw	Amide Proton Transfer-weighted
RF	Radio Frequency
SD	Standard Deviation
CI	Confidence Interval
N_p_	Number of Pulses
B_1,avg_	Average B_1_
HGG	High-Grade Glioma
LGG	Low-Grade Glioma
PCA	Principal Component Analysis
CEST	Chemical Exchange Saturation Transfer
AUC	Area Under the Curve
ROC	Receiver Operating Characteristic
SE	Spin-Echo
TSE	Turbo Spin-Echo
FSE	Fast Spin-Echo
GRE	Gradient Echo
EPI	Echo-Planar Imaging
GRASE	Gradient and Spin Echo
SPACE	Sampling Perfection with Application optimized Contrasts using different flip angle Evolution
