# Supplementary material for: Quantitative Consistency of Amide Proton Transfer-Weighted MRI for Brain Tumor Differentiation: Systematic Review of Clinical Evidence
Source: Tomography. 2026 May 6;12(5):65. doi: 10.3390/tomography12050065 (PMC13210856; doi:10.3390/tomography12050065)

Supplementary Materials

The literature search was based around three terms: 1. Amide Proton Transfer imaging, 2. Gliomas, and 3. Grading. The search was carried out in a systematic manner using these three terms and synonyms along with their intersect. The following search terms were used:

Term 1: 'amide proton transfer' OR ('apt' AND (amide OR cest OR 'chemical exchange' OR 'proton transfer')) OR (amide AND (cest OR 'chemical exchange' OR 'magnetization transfer' OR 'proton transfer'))

Term 2: glioma OR glioblastoma OR ((gbm OR hgg OR lgg) AND (tumor OR tumour)) OR (('brain' OR neural OR cns OR cerebral) AND (tumor OR tumour OR cancer OR neoplasm OR astrocytoma))

Term 3: 'high grade' OR 'low grade' OR grade OR grading OR malignant OR malignancy

Principal Component Analysis

Principal component analysis was performed on each parameter group defined to identify the dominant eigenvector explaining the largest proportion of parameter variance across studies. The primary principal component of parameters relating to exchange rate tuning explained 40% of the variance across this group of parameters. This component aligned along a 0.36 μT increase in B_1, a 0.5 s increase in T_{sat}, and a 7.9% decrease in DC. While higher saturation power and lower duty cycle favor faster or broader exchange processes, extended saturation duration tends to favor slower exchange mechanisms. The primary PC of read-out parameters explained 34% of the variance across this group and aligned along a 4.6% increase in Phillips scanner usage, a 2.8% decrease in 3D imaging sequences, a 21.3% increase in spin-echo vs. gradient-echo usage, a 1289 ms increase in TR, and a 1055 ms increase in recovery delay time. In contrast, a 6.6% decrease in Siemens usage and a 4.6% decrease in GE usage showed an opposing trend. For steady-state parameters, 67% of variance aligned with a 478.6 ms increase in saturation duration corresponding with a 1162.4 ms increase in relaxation delay—increases in both the saturation duration and relaxation delay approach conditions for the steady state. When PCA was applied to all parameters together, 27.8% of variance was explained by a pattern including: 0.3 μT increase in B_1, 261 ms increase in in T_{sat}, 727 ms increase in TR, 1.4% decrease in DC, 20.2% increase in 2D vs 3D, 8.6% increase in spin-echo vs gradient-echo, 20.9% increase in groups using GE, 1.6% decrease in groups using Siemens, and a 19.7% decrease in groups using Philips-based sequences.

Across the imaging parameters included in this study, 9 quantitative input variables were examined, comprising 4 continuous variables (B_0, B_1, TR, and T_{sat}) and 5 categorical variables (1 dummy variable for each vendor, 1 dummy variable specifying Spin-Echo vs Gradient Echo, and 1 variable specifying 2D vs 3D acquisition). Out of the 31 studies, only 22 of them fully reported all the scan parameters that were examined. With 27.8% of variance being described along the primary principal eigenvector across the entire parameter group, this indicated that there was a minor amount of consensus in the changes in parameters across studies. This fact is understandable as the number and diversity of all scan parameters does not necessarily warrant a uniform strategy for parameter decision. On the other hand, the two parameters that push CEST contrast toward the steady state,

saturation time and relaxation delay time, displayed 67% of its variance across a principal component eigenvector that increases both parameters.

Among exchange rate tuning parameters, 40% of variance was explained by the first principal component, which corresponded to increased B_1 and saturation duration but decreased duty cycle. This pattern reflects a mixed sensitivity profile: while increased B_1 and reduced DC tune the sequence toward faster and broader exchange processes, the accompanying increase in saturation duration biases the contrast toward slower exchange regimes, closer to steady state. These conflicting influences suggest that the primary design goal may be to enhance signal sensitivity, even at the expense of exchange specificity. This raises concerns about potential signal contamination from faster-exchanging amine protons, which may reduce the specificity of APT for amide signals. For the magnetization recovery group, 34% of variance aligned with the first PC, largely reflecting changes in repetition time, relaxation delay, and readout type. Although vendor preference was a contributing variable, its overall effect size was limited, suggesting that timing parameters dominated this component.

Table S1. Exchange Principal Component: Exchange Rate Tuning

Authors	PC1	PC2	PC3
1. Zhou et al. (2013)	-1.67	-0.73	1.19
2. Park et al. (2015)	-2.46	-0.81	0.62
3. Park et al. (2015)	-2.46	-0.81	0.62
4. Sakata et al. (2015)	0.96	-2.11	-1.04
5. Togao et al. (2016)	0.73	0.86	0.55
6. Bai et al. (2017)	1.27	-2.00	-0.69
7. Choi et al. (2017)	-0.22	0.51	-0.53
8. Sakata et al. (2017)	-1.18	-0.34	0.61
9. Su et al. (2017)	-0.54	0.39	-0.89
10. Zou et al. (2017)	-0.09	0.31	-0.53
11. Chen et al. (2018)	-0.88	0.14	-0.34
12. Paech et al. (2018)	2.14	-1.37	3.02
13. Sakata et al. (2018)	0.96	-2.11	-1.04
14. Zhang et al. (2018)	-0.54	0.39	-0.89
15. Durmo et al. (2020)	0.64	-1.19	-0.78
16. Kang et al. (2020)	-0.54	0.39	-0.89
17. Su et al. (2020)	-0.38	0.45	-0.71
18. Debnath et al. (2021)	-0.21	0.50	-0.53
19. Su et al. (2021)	0.41	0.74	0.19
20. Su et al. (2021)	0.73	0.86	0.55
21. Xu et al. (2021)	0.73	0.86	0.55
22. Guo et al. (2022)	0.46	0.92	-0.93
23. Hou et al. (2022)	-0.22	0.51	-0.53
24. Liu et al. (2022)	0.73	0.86	0.55
25. Zhang et al. (2022)	*NaN*	*NaN*	*NaN*
26. Filimonova et al. (2024)	*NaN*	*NaN*	*NaN*
27. Hou et al. (2024)	0.73	0.86	0.55
28. Ying et al. (2025)	-1.37	-0.30	1.09
29. Yegnaraman et al. (2025)	0.73	0.86	0.55
30. Takami et al. (2025)	0.73	0.86	0.55
31. Jiang et al. (2025)	0.78	0.48	-0.89

Eigenvectors		
v_1	v_2	v_3
0.62	0.41	-0.67
0.64	0.24	0.73
-0.46	0.88	0.11
**Eigenvalues**		
λ_1	λ_2	λ_3
1.19	0.95	0.86

Eigenvectors (units)			
	v_1	v_2	v_3
B_1 (μT)	0.36	0.24	-0.40
T_{sat} (s)	0.52	0.19	0.59
DC (%)	-7.88	15.15	1.96

Table S2. Read-Out Principal Component: Magnetization Recovery

Authors	PC1	PC2	PC3	PC4	PC5	PC6	PC7
1. Zhou et al. (2013)	-0.24	-1.64	-0.77	-0.30	-0.01	-0.04	0.05
2. Park et al. (2015)	-2.27	-1.75	-1.17	-0.19	-0.23	-0.22	0.10
3. Park et al. (2015)	-2.27	-1.75	-1.17	-0.19	-0.23	-0.22	0.10
4. Sakata et al. (2015)	*NaN*	*NaN*	*NaN*	*NaN*	*NaN*	*NaN*	*NaN*
5. Togao et al. (2016)	1.21	-0.56	-0.44	1.02	-0.82	0.48	-0.09
6. Bai et al. (2017)	-0.92	1.87	-0.65	-0.62	-0.38	0.10	0.05
7. Choi et al. (2017)	0.25	-1.75	-0.49	0.39	0.54	-0.08	0.11
8. Sakata et al. (2017)	3.96	0.97	0.66	-0.85	-1.19	-0.70	0.17
9. Su et al. (2017)	*NaN*	*NaN*	*NaN*	*NaN*	*NaN*	*NaN*	*NaN*
10. Zou et al. (2017)	0.34	-0.68	-0.51	1.46	-0.65	0.27	0.21
11. Chen et al. (2018)	-2.28	0.22	2.09	-0.13	-1.51	0.30	0.10
12. Paech et al. (2018)	*NaN*	*NaN*	*NaN*	*NaN*	*NaN*	*NaN*	*NaN*
13. Sakata et al. (2018)	*NaN*	*NaN*	*NaN*	*NaN*	*NaN*	*NaN*	*NaN*
14. Zhang et al. (2018)	0.12	1.65	-0.08	0.73	0.70	-0.03	0.35
15. Durmo et al. (2020)	*NaN*	*NaN*	*NaN*	*NaN*	*NaN*	*NaN*	*NaN*
16. Kang et al. (2020)	0.12	1.65	-0.08	0.73	0.70	-0.03	0.35
17. Su et al. (2020)	-1.02	0.79	-0.63	-1.69	0.81	-0.29	0.07
18. Debnath et al. (2021)	NaN	NaN	NaN	NaN	NaN	NaN	NaN
19. Su et al. (2021)	-1.25	1.82	-0.68	-0.44	-0.29	0.15	-0.19
20. Su et al. (2021)	1.78	1.89	0.04	-0.13	0.36	0.29	-0.02
21. Xu et al. (2021)	-0.76	1.89	-0.64	-0.69	-0.39	0.24	-0.27
22. Guo et al. (2022)	-0.55	-0.95	2.73	-0.25	0.60	0.04	-0.01
23. Hou et al. (2022)	NaN	NaN	NaN	NaN	NaN	NaN	NaN
24. Liu et al. (2022)	1.68	-1.55	-0.38	-0.35	0.25	0.18	-0.14
25. Zhang et al. (2022)	NaN	NaN	NaN	NaN	NaN	NaN	NaN
26. Filimonova et al. (2024)	NaN	NaN	NaN	NaN	NaN	NaN	NaN
27. Hou et al. (2024)	1.68	-1.55	-0.38	-0.35	0.25	0.18	-0.14
28. Ying et al. (2025)	-0.53	0.34	0.30	1.52	-0.18	-1.00	-0.38
29. Yegnaraman et al. (2025)	1.93	-1.52	-0.36	-0.47	0.19	0.20	-0.12
30. Takami et al. (2025)	-0.40	1.58	-0.13	1.03	0.85	0.14	-0.26
31. Jiang et al. (2025)	-0.58	-0.96	2.72	-0.23	0.61	0.05	-0.05

Table S2 (cont). Read-Out Principal Component cont.

Eigenvectors						
v_1	v_2	v_3	v_4	v_5	v_6	v_7
-0.05	-0.55	0.01	-0.54	0.61	-0.17	-0.06
0.46	-0.11	0.26	0.66	0.52	-0.03	0.04
0.61	0.09	0.04	-0.29	-0.10	0.31	-0.65
0.60	0.08	0.06	-0.34	-0.17	-0.18	0.67
-0.09	0.59	-0.22	-0.17	0.53	0.48	0.21
-0.19	-0.11	0.83	-0.13	-0.09	0.47	0.15
0.09	-0.56	-0.43	0.16	-0.15	0.62	0.23
**Eigenvalues**						
λ_1	Λ_2	λ_3	λ_4	λ_5	λ_6	λ_7
2.41	2.15	1.23	0.63	0.43	0.11	0.04

	Eigenvectors (units)						
	v_1	v_2	v_3	v_4	v_5	v_6	v_7
**3D (%)**	-2.75	1.51	0.01	-0.01	0.00	0.00	0.00
**Spin Echo (%)**	21.29	-2.25	-0.59	-0.39	-0.20	0.01	0.00
**TR (s)**	1.29	0.12	0.00	0.00	0.00	0.00	0.00
**T_{rec} (s)**	1.06	0.08	0.00	0.00	0.00	0.00	0.00
**GE (%)**	-4.55	-2.69	0.60	-0.10	-0.05	-0.03	-0.01
**SIEGY (%)**	-6.60	0.70	0.58	-0.08	0.01	0.00	0.00
**PHG (%)**	4.59	-2.57	1.12	0.17	-0.03	-0.02	0.00

T_{rec} (magnetization recovery time) = TR - T_{sat}

Table S3. Steady-state Principal Component

Authors	PC1	PC2
1. Zhou et al. (2013)	-0.39	-0.14
2. Park et al. (2015)	-2.10	-0.05
3. Park et al. (2015)	-2.10	-0.05
4. Sakata et al. (2015)	*NaN*	*NaN*
5. Togao et al. (2016)	1.21	0.75
6. Bai et al. (2017)	-0.20	0.02
7. Choi et al. (2017)	-0.41	-0.19
8. Sakata et al. (2017)	2.17	-2.73
9. Su et al. (2017)	-0.66	-0.78
10. Zou et al. (2017)	-0.39	-0.14
11. Chen et al. (2018)	-1.31	0.08
12. Paech et al. (2018)	*NaN*	*NaN*
13. Sakata et al. (2018)	*NaN*	*NaN*
14. Zhang et al. (2018)	-0.66	-0.78
15. Durmo et al. (2020)	*NaN*	*NaN*
16. Kang et al. (2020)	-0.66	-0.78
17. Su et al. (2020)	-0.54	-0.48
18. Debnath et al. (2021)	-0.41	-0.18
19. Su et al. (2021)	0.10	1.01
20. Su et al. (2021)	1.85	0.10
21. Xu et al. (2021)	0.78	1.17
22. Guo et al. (2022)	-0.28	0.11
23. Hou et al. (2022)	-0.41	-0.19
24. Liu et al. (2022)	1.60	0.35
25. Zhang et al. (2022)	*NaN*	*NaN*
26. Filimonova et al. (2024)	*NaN*	*NaN*
27. Hou et al. (2024)	1.60	0.35
28. Ying et al. (2025)	-0.71	0.54
29. Yegnaraman et al. (2025)	1.77	0.19
30. Takami et al. (2025)	0.37	1.59
31. Jiang et al. (2025)	-0.22	0.26

Eigenvectors	
v_1	v_2
0.71	-0.71
0.71	0.71
**Eigenvalues**	
λ_1	λ_2
1.34	0.66

Eigenvectors (units)		
	v_1	v_2
T_{rec} (s)	1.16	1.16
T_{sat} (s)	0.48	-0.48

Table S4. Total Principal Component: Total Parameters

Authors	PC1	PC2	PC3	PC4	PC5	PC6	PC7	PC8	PC9
1. Zhou et al. (2013)	-2.34	0.29	-0.95	-0.32	-0.24	-0.31	0.35	0.47	0.40
2. Park et al. (2015)	-3.52	-1.13	-0.81	0.08	0.31	-0.05	0.01	0.06	0.08
3. Park et al. (2015)	-3.52	-1.13	-0.81	0.08	0.31	-0.05	0.01	0.06	0.08
4. Sakata et al. (2015)	NaN	NaN	NaN	NaN	NaN	NaN	NaN	NaN	NaN
5. Togao et al. (2016)	0.59	1.77	-0.57	-0.02	-0.29	-0.29	-1.25	-0.35	0.03
6. Bai et al. (2017)	1.60	-2.99	-0.73	-3.31	-1.27	0.25	-0.12	0.13	-0.01
7. Choi et al. (2017)	-1.18	1.19	0.14	-0.28	0.25	1.13	-0.08	-0.25	-0.23
8. Sakata et al. (2017)	0.72	0.89	-0.78	0.80	-2.16	-0.96	1.49	-0.53	-0.10
9. Su et al. (2017)	NaN	NaN	NaN	NaN	NaN	NaN	NaN	NaN	NaN
10. Zou et al. (2017)	-0.33	0.62	-0.28	0.10	-0.76	0.84	-1.22	-0.73	-0.09
11. Chen et al. (2018)	-0.84	-1.87	2.16	0.69	-0.37	-1.33	-0.71	-0.61	0.16
12. Paech et al. (2018)	NaN	NaN	NaN	NaN	NaN	NaN	NaN	NaN	NaN
13. Sakata et al. (2018)	NaN	NaN	NaN	NaN	NaN	NaN	NaN	NaN	NaN
14. Zhang et al. (2018)	1.02	-1.01	-0.11	1.15	-0.19	1.33	0.23	-0.21	0.15
15. Durmo et al. (2020)	NaN	NaN	NaN	NaN	NaN	NaN	NaN	NaN	NaN
16. Kang et al. (2020)	1.02	-1.01	-0.11	1.15	-0.19	1.33	0.23	-0.21	0.15
17. Su et al. (2020)	-0.03	-1.44	-0.04	-0.12	1.59	0.31	1.28	-0.34	-0.28
18. Debnath et al. (2021)	NaN	NaN	NaN	NaN	NaN	NaN	NaN	NaN	NaN
19. Su et al. (2021)	1.30	-1.38	-0.58	0.28	1.19	-0.67	-0.33	-0.20	-0.17
20. Su et al. (2021)	2.47	0.58	-0.59	0.54	0.11	-0.28	0.37	0.23	0.38
21. Xu et al. (2021)	1.68	-0.96	-0.71	0.12	1.25	-1.09	-0.26	-0.11	-0.10
22. Guo et al. (2022)	-0.07	0.43	3.21	-0.01	0.12	0.10	0.29	0.19	-0.01
23. Hou et al. (2022)	NaN	NaN	NaN	NaN	NaN	NaN	NaN	NaN	NaN
24. Liu et al. (2022)	-0.04	2.44	-0.24	-0.75	0.44	-0.12	0.10	0.03	-0.03
25. Zhang et al. (2022)	NaN	NaN	NaN	NaN	NaN	NaN	NaN	NaN	NaN
26. Filimonova et al. (2024)	NaN	NaN	NaN	NaN	NaN	NaN	NaN	NaN	NaN
27. Hou et al. (2024)	-0.04	2.44	-0.24	-0.75	0.44	-0.12	0.10	0.03	-0.03
28. Ying et al. (2025)	-0.54	-0.39	-0.45	1.36	-1.23	-0.21	-0.38	1.09	-0.50
29. Yegnaraman et al. (2025)	0.03	2.52	-0.27	-0.77	0.40	-0.18	0.20	-0.05	0.00
30. Takami et al. (2025)	1.91	-0.12	-0.35	0.69	0.53	0.24	-0.55	0.91	0.13
31. Jiang et al. (2025)	0.09	0.25	3.15	-0.71	-0.23	0.13	0.22	0.40	0.01

Table S4 (cont). Total Principal Component cont.

Eigenvectors								
v_1	v_2	v_3	v_4	v_4	v_6	v_7	v_8	v_9
-0.40	0.25	0.20	-0.35	0.43	0.16	0.57	0.29	-0.07
0.18	0.49	0.14	0.25	-0.40	0.56	-0.02	0.39	0.14
0.34	0.43	-0.15	-0.09	-0.25	-0.32	0.56	-0.41	0.15
0.42	-0.36	-0.22	0.05	0.40	0.28	0.20	0.09	0.59
-0.04	-0.07	0.80	-0.01	-0.09	-0.30	-0.07	-0.02	0.50
-0.39	0.38	-0.26	-0.26	0.11	0.16	-0.41	-0.35	0.49
0.44	0.13	0.39	-0.23	0.32	0.37	-0.19	-0.45	-0.33
0.38	0.38	-0.10	-0.20	0.31	-0.46	-0.34	0.49	-0.01
-0.13	0.28	0.05	0.80	0.46	-0.12	0.03	-0.17	-0.03
Eigenvalues								
λ_1	Λ_2	λ_3	λ_4	λ_5	λ_6	λ_7	λ_8	λ_9
2.50	2.23	1.45	0.95	0.73	0.50	0.41	0.20	0.04

	Eigenvectors (units)								
	v_1	v_2	v_3	v_4	v_4	v_6	v_7	v_8	v_9
**3D (%)**	-20.18	12.56	10.27	-17.92	22.00	8.08	28.84	14.61	-3.38
**Spin Echo (%)**	8.56	23.00	6.64	11.79	-18.77	26.56	-0.80	18.58	6.76
**TR (s)**	0.73	0.90	-0.31	-0.19	-0.53	-0.67	1.18	-0.86	0.33
**GE (%)**	20.90	-17.83	-10.64	2.57	19.57	13.65	10.09	4.64	29.27
**SIEGY (%)**	-1.56	-2.52	27.99	-0.19	-3.11	-10.62	-2.38	-0.55	17.70
**PHG (%)**	-19.68	19.29	-13.29	-13.27	5.59	8.14	-20.54	-17.42	24.45
**B_1 (μT)**	0.28	0.08	0.25	-0.15	0.21	0.24	-0.12	-0.29	-0.21
**T_{sat} (s)**	0.26	0.26	-0.07	-0.14	0.21	-0.32	-0.23	0.33	-0.01
**DC (%)**	-1.38	3.01	0.51	8.57	4.95	-1.24	0.33	-1.82	-0.30

T_{rec} (magnetization recovery time) = TR - T_{sat}

Figure S1. QUADAS-2: Quality Assessment Results.

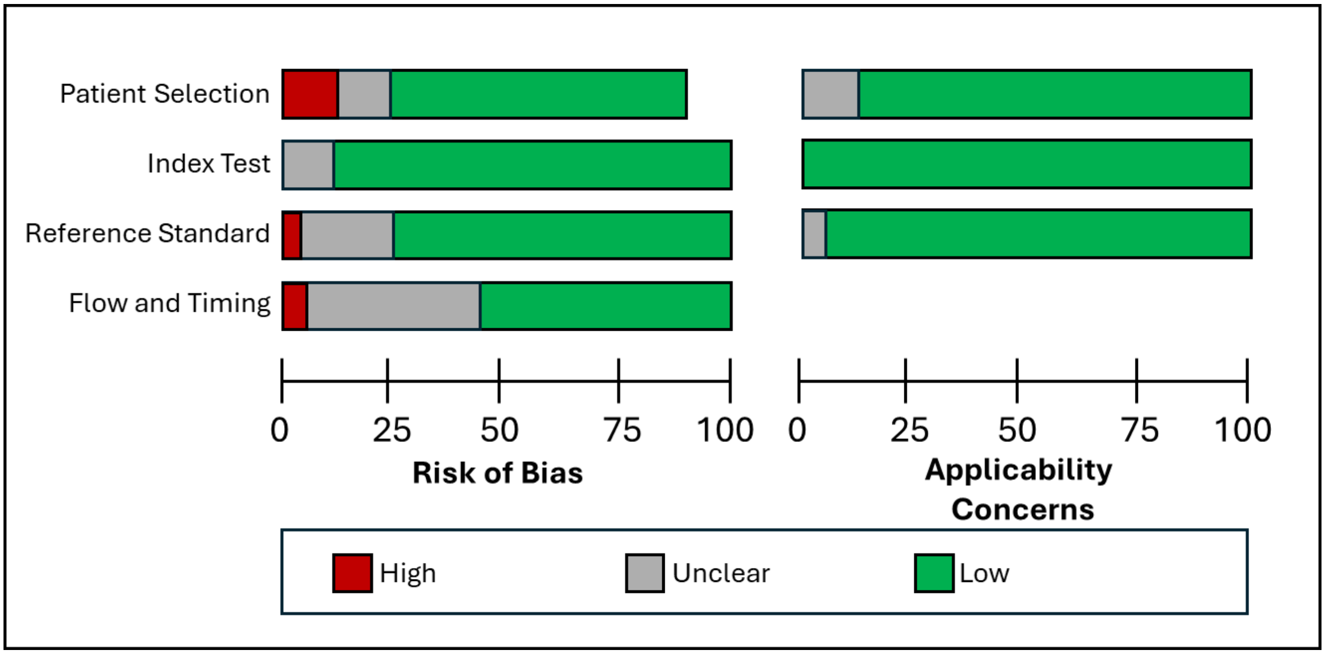

Figure S2. Forest plot of logit(AUC) comparing LGG to HGG among 31 studies.

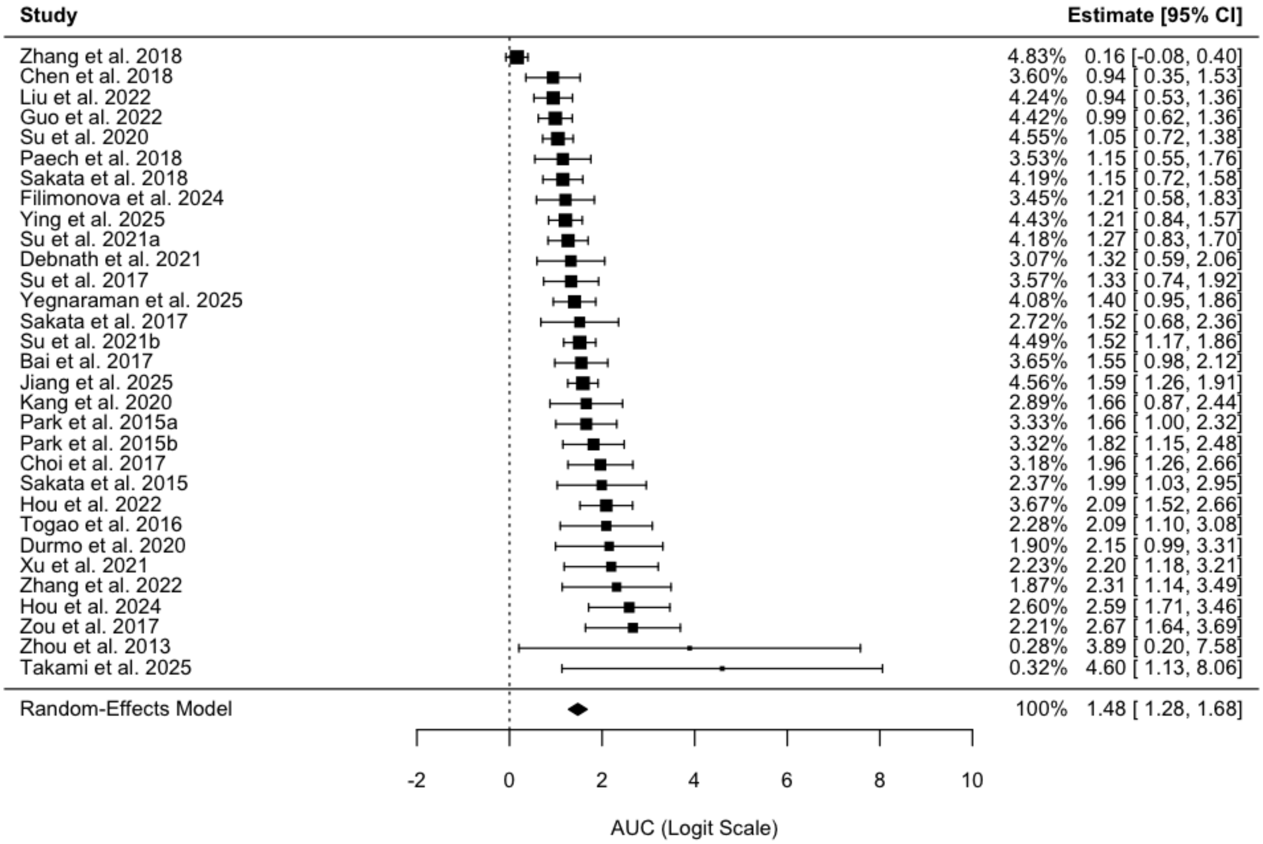

Supplement: Supplementary file 1 [file tomography-12-00065-s001.zip › supplementary.pdf]
